# Frequency Dependence of Microwave Permeability in Composite Materials Filled with Gadolinium Particles: Effects of Temperature and Filler Fraction

**DOI:** 10.3390/s26092767

**Published:** 2026-04-29

**Authors:** Ilya V. Komarov, Nikita A. Buznikov, Sergey S. Maklakov, Sergey N. Starostenko, Svetlana F. Lomaeva, Galina V. Kurlyandskaya

**Affiliations:** 1 Institute for Theoretical and Applied Electromagnetics, Russian Academy of Sciences, 125412 Moscow, Russia; komarov@itae.ru (I.V.K.); n_buznikov@mail.ru (N.A.B.); maklakov@itae.ru (S.S.M.); snstar@mail.ru (S.N.S.); 2 Physical-Technical Institute, Udmurt Federal Research Center, Ural Branch of the Russian Academy of Sciences, 426067 Izhevsk, Russia; lomayevasf@mail.ru; 3 Institute of Natural Sciences and Mathematics, Ural Federal University, 620002 Ekaterinburg, Russia

**Keywords:** gadolinium, microwave permeability, Curie temperature, mixing rules, intrinsic permeability, temperature-tunable microwave devices

## Abstract

The microwave permeability of composites containing microsized gadolinium powder in paraffin wax was studied as a function of frequency, temperature and gadolinium fraction for a series of composites with a powder volume fraction of 5% to 50%. The constitutive parameters of the composites were measured by the Nicolson–Ross–Weir technique in a standard 7 × 3 mm coaxial line in the frequency range of 0.1 to 10 GHz and the temperature range of 5 to 26 °C. In the indicated ranges, the permittivity was independent of frequency and temperature, and the real part of the permittivity only depended on the gadolinium powder volume fraction. It was found that the peak of the magnetic losses shifts towards lower frequencies with an increase in temperature. The Curie temperature of the composites found from the temperature dependence of the static permeability was close to 15 °C, being practically independent of the gadolinium volume fraction. To retrieve the intrinsic permeability of the filler particles, the Odelevsky mixing rule was used. The measured dependence of the static permittivity on the gadolinium volume fraction was in good agreement with the fitting by the Odelevsky mixing rule. Using the values of the percolation threshold and the depolarization factor obtained as a result of the fitting of the static permittivity, the frequency dependence of the intrinsic permeability of the filler particles was retrieved. The temperature effect on the intrinsic permeability was also analyzed. The obtained results may be useful for designing microwave devices, including temperature-tunable microwave screens and sensors.

## 1. Introduction

Gadolinium has attracted considerable attention from researchers due to its unique physical properties and a wide range of promising applications. Gadolinium is the only pure metal that undergoes a ferromagnetic–paramagnetic phase transition at a Curie temperature close to room temperature [1,2]. For single-crystal and polycrystalline gadolinium bulk samples, the Curie temperature is in the range of 291 to 294 K [3,4,5,6,7,8,9,10]. Note that the measured values of the Curie temperature of gadolinium depend on the shape and crystal structure of the studied sample, as well as on the content of impurities in gadolinium [7,11,12]. From a fundamental point of view, gadolinium serves as an ideal material for studying the nature of the ferromagnetic–paramagnetic phase transition. On the other hand, gadolinium and its alloys are well-known materials used in magnetic cooling refrigerators working at room temperature [13,14,15,16]. The operating principle is based on the magnetocaloric effect (MCE), i.e., the reversible change in the temperature of a magnetic material under the application of a magnetic field, which has been the subject of intensive recent research [17]. The MCE has been studied in bulk gadolinium, rapidly quenched ribbons, gadolinium flakes, and multilayered materials, where the nanostructuring procedure allows the field dependence of the MCE to be controlled [18]. Special attention has been paid to the comparison of the results obtained for gadolinium nanostructured materials with bulk states, revealing the importance of size effects.

In addition to applications in magnetic cooling refrigerators, gadolinium powders show great potential as fillers in magnetic composites. Composite materials with ferromagnetic fillers have attracted attention due to their enormous fundamental and practical interest [19,20,21]. These composites exhibit promising magnetic, electrical and mechanical properties, which cannot be achieved in single-phase materials. Filled magnetic composites are useful in the development of various high-frequency devices, such as antennas (see, for example, [21] and references therein), sensors [22,23,24], electromagnetic interference shielding [25,26,27] and microwave absorbers [28,29,30,31,32]. The microwave performance of these devices is governed by the properties of ferromagnetic particles, particularly saturation magnetization and permeability. Since the saturation magnetization of gadolinium is approximately 15% higher than that of iron in the low-temperature range, gadolinium has several advantages for use in microwave magnetic composites under particular application conditions.

However, there are very few studies devoted to the microwave properties of composites with gadolinium powder. The development of composite materials for various microwave applications requires knowledge of the intrinsic permeability of the ferromagnetic filler. This can be done using the ferromagnetic resonance (FMR) phenomenon. Previously, FMR measurements were carried out at a frequency of 9 GHz for paraffin-bound composites containing gadolinium nanoparticles with a volume fraction below 1% [33,34]. The microwave behavior of paraffin-bound composites filled with microsized gadolinium powder at a 30% volume fraction was studied in [35]. The dependences of microwave permeability on frequency and temperature were obtained, and the characteristics of the composite as a temperature-tunable microwave screen were analyzed. Although real technological applications most probably require additional studies of the interactions between the polymer matrix and magnetic particles, it is precisely the study of the high-frequency properties of the magnetic particles in the paraffin matrix that allows the filler itself to be most accurately characterized. Other fillers, in addition to introducing additional elastic stresses, can also cause chemical reactions with the matrix material.

In this work, the microwave properties of a series of paraffin-bound composites with a gadolinium powder volume fraction of 5% to 50% were studied. The constitutive parameters of the composites were measured in the frequency range of 0.1 to 10 GHz and the temperature range of 5 to 26 °C. The Curie temperature of the composites was estimated from the temperature dependence of the static permeability. Here, the frequency dependence of the intrinsic permeability of the filler particles was retrieved from the measured permeability of the composites using the Odelevsky mixing rule. The temperature effect on the intrinsic permeability was analyzed.

## 2. Materials and Methods

The gadolinium powder was obtained from a commercial ingot (Ural-Metal, Ekaterinburg, Russia), which was mechanically crushed into pieces approximately 0.5 mm thick. Then, the resulting pieces were ground in a planetary mill in an argon medium to prevent ignition. Details of the synthesis and analysis of the microstructure and morphology of the powder were presented in [35]. Energy-dispersive X-ray spectroscopy showed traces of surface oxidation of the powder. X-ray phase analysis showed that the obtained powder was metallic gadolinium containing approximately 10% vol. of gadolinium hydride. An increase in the milling time leads to further partial transformation of the metal into gadolinium hydride [35]. Although the filler is mainly represented by gadolinium microparticles, it is understandable that micro- and nanocomponent materials of highly reactive elements cannot be represented by pure 100% metallic phase due to surface oxidation or formation of a surface layer having properties different from those of the main material phase [36,37]. Therefore, we calculate the internal permeability of the powder particles rather than that of pure gadolinium. To avoid misunderstanding, the term “the intrinsic permeability of filler particles” is used in order to emphasize that the material under study was metallic gadolinium containing approximately 10% vol. of gadolinium hydride.

The paraffin-bound composite of the gadolinium powder was prepared by mixing it in an ultrasonic bath heated up to 100 °C. A series of composites with a powder volume fraction of 5% to 50% were obtained.

**Figure 1:**
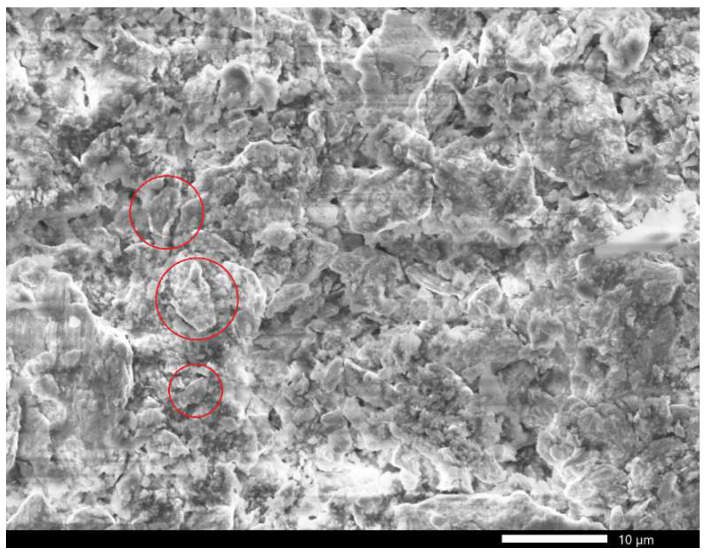
SEM image of a composite with gadolinium powder. Red circles indicate large gadolinium particles.

The composite microstructure was analyzed using JEOL-7000 scanning electronic microscope (SEM) (JEOL Ltd., Tokyo, Japan). Figure 1 shows an SEM image of a paraffin-bound composite. The gadolinium powder consists of particles of various shapes and sizes. The largest gadolinium particles have a stone-like shape, and the average size of these particles ranges from 2 to 5 μm.

The frequency dependences of the microwave permittivity *ε* and microwave permeability *μ* of the composites were measured by the Nicolson–Ross–Weir technique [38,39] in a standard 7 × 3 mm coaxial line in the frequency range of 0.1 to 10 GHz by a Keysight N5224B vector network analyzer (Keysight Technologies, Colorado Springs, CO, USA). For the microwave measurements, composite samples were pressed into disks with a thickness of 1.5–4.5 mm. The static permittivity and permeability were defined as the median values in the frequency range of 100 to 200 MHz.

Most microwave permeability measurements were carried out in the absence of an external magnetic field. Additionally, the microwave permeability of the composite with a gadolinium volume fraction of 25% was measured under a static magnetic field at some temperatures. For these measurements, the coaxial line was placed inside a coil producing a magnetic field of up to 800 Oe. The external static magnetic field was applied along the axis of the measurement line and perpendicular to the microwave magnetic field, as established for FMR conditions.

**Figure 2:**
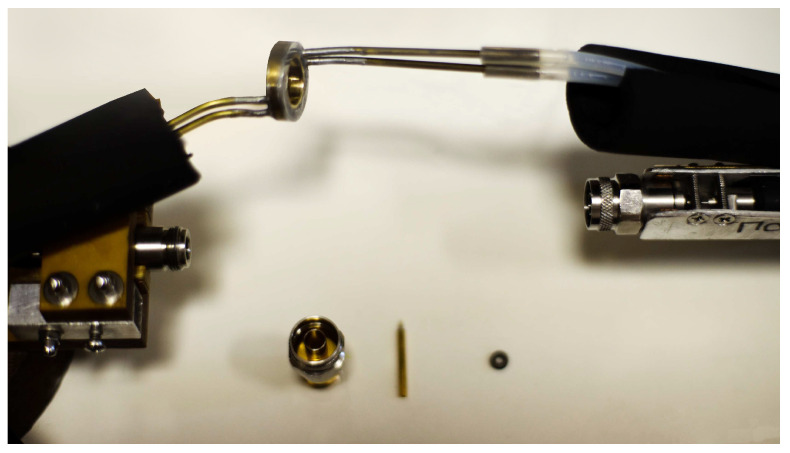
Image of a microwave measuring line with external cooling.

To study the temperature dependence of the microwave properties of the composites, a cooling jacket was constructed for the measuring line. An image of the measuring line is shown in Figure 2. The cooling was achieved by a flow of a mixture of ethyl and isopropyl alcohols through the jacket. To reduce the air gap and increase thermal conductivity, a thermally conductive paste was applied to the cell–jacket. The temperature was measured with a type K thermocouple attached to the outside of the cell close to the studied sample. The coolant temperature was controlled using an LOIP FT-311-80 liquid cryostat (AO LOIP, St. Petersburg, Russia).

Microwave measurements of composites with different volume fractions of gadolinium powder were performed in the temperature range of 5 to 26 °C. In this temperature range, approximately 70 measurements were carried out for each composite sample. In the temperature range of 10 to 20 °C, the measurements were performed at every 0.2 °C intervals. To reduce the temperature gradient in the sample, a holding time of approximately 2 min was allowed at each temperature point.

For analysis, the measured frequency dependence of the permeability of composites was fitted by a sum of two Lorentzian terms [19,40]:

(1) $$\mu =1+\sum_{j=1}^{2}\frac{4\pi \chi_{s,j}}{1-i\beta_{j}f/f_{\mathrm{res},j}-{\left(f/f_{\mathrm{res},j}\right)}^{2}},$$

where *χ*_s,*j*_, *f*_res,*j*_ and *β_j_* are the partial static susceptibility, resonance frequency and damping factor of the *j*-th resonance (*j* = 1, 2), respectively.

To find the parameters in the Lorentzian frequency dispersion law, the least mean square deviation of the calculated permeability from the measured one was minimized. The minimization was performed using numerical methods.

## 3. Results and Discussion

### 3.1. Frequency Dependence of Composite Permeability

In the studied frequency range, the real part of the permittivity of the composites remains almost constant, while the imaginary part is close to zero. The real part of the permittivity depends only on the gadolinium powder volume fraction and may be considered as the static permittivity.

**Figure 3:**
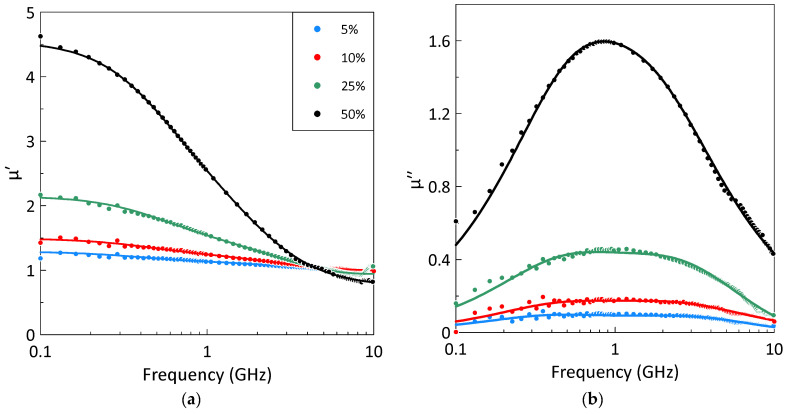
The measured frequency dependences of the real (**a**) and imaginary (**b**) parts of the composite permeability at 10 °C for different volume fractions of gadolinium particles. Symbols represent experimental data; lines correspond to the fitting by the Lorentzian dispersion law, Equation (1). The legend describing the different gadolinium volume fractions is the same for parts (**a**,**b**).

The frequency dependences of the permeability of the composites at different powder volume fractions measured at 10 °C are shown in Figure 3. The static permeability and magnetic loss increase with the gadolinium volume fraction.

The frequency dependence of the imaginary part of the permeability for all composites exhibits a broad maximum, which is fitted with good accuracy by a sum of two Lorentzian terms, Equation (1). Analysis showed that the magnetic losses in the studied composites consist of a lower-frequency resonance at approximately 0.6 GHz and a higher-frequency resonance near 1.1 GHz. The position of the magnetic loss peak depends weakly on the volume fraction of gadolinium.

**Figure 4:**
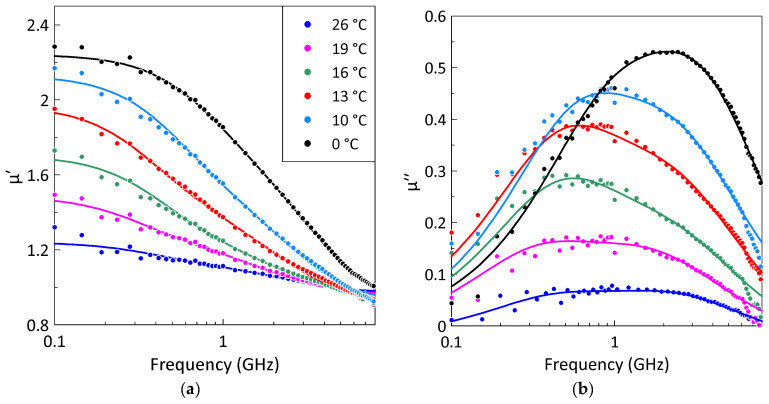
The measured frequency dependences of the real (**a**) and imaginary (**b**) parts of the composite permeability for a gadolinium particle volume fraction of 25% at different temperatures. Symbols represent experimental data; lines correspond to the fitting by the Lorentzian dispersion law, Equation (1). The legend describing the different temperatures is the same for parts (**a**,**b**).

Figure 4 shows the frequency dependence of the permeability of composites with a 25% volume fraction of gadolinium particles at different temperatures. The static permeability decreases with increasing temperature. It follows from Figure 4 that, with a growth in temperature, the peak of magnetic losses shifts towards lower frequencies and broadens. At the temperatures above 16 °C, the magnetic losses drop sharply, which may indicate a ferromagnetic–paramagnetic phase transition in the gadolinium particles. The broadening of the peak can be connected with the existence of a certain size distribution of gadolinium particles, affecting the Curie temperature of individual particles.

**Figure 5:**
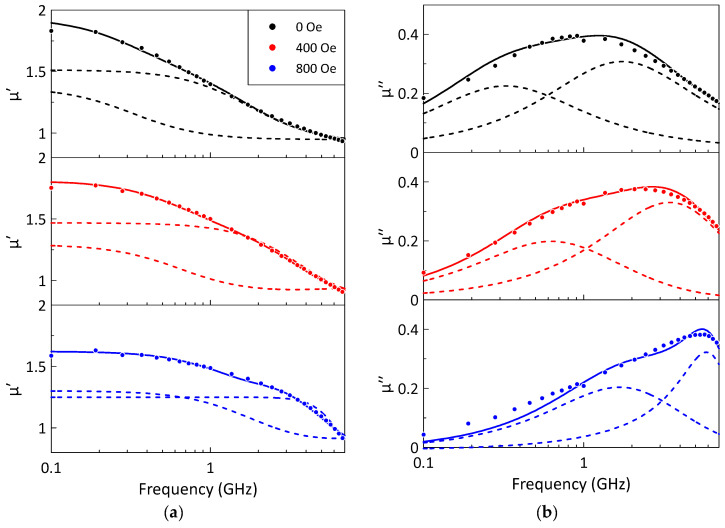
The measured frequency dependences of the real (**a**) and imaginary (**b**) parts of the composite permeability for a gadolinium particle volume fraction of 25% at 10 °C and different values of the static magnetic field. Symbols represent experimental data; solid lines correspond to the fitting by the Lorentzian dispersion law, Equation (1); dashed lines represent two Lorentzian resonances. The legend describing the different static magnetic fields is the same for parts (**a**,**b**).

To analyze the nature of the observed two peaks in magnetic losses and the physical mechanisms leading to the complex frequency dependence of the microwave permeability, the constitutive parameters of the composites were measured under the application of astatic magnetic field. Figure 5 shows the frequency dependence of the microwave permeability for a composite with a gadolinium volume fraction of 25% at a temperature of 10 °C and different values of the external static magnetic field.

The application of a static magnetic field changes the shape of the magnetic loss peak. The magnitude of the lower-frequency maximum decreases, and the position of the maximum shifts towards higher frequencies (Figure 5b). At the same time, the higher-frequency maximum also shifts towards higher frequencies but maintains a nearly constant magnitude. Such dependences may be associated with changes in the magnetic structure of the filler particles with increasing static magnetic field. Based on the observed frequency dependence of the microwave permeability in the presence of a static magnetic field, it can be concluded that the higher-frequency maximum is due to FMR, while the lower-frequency maximum corresponds to non-resonant microwave phenomena [41,42] and can be associated with the magnetic domain structure of the filler particles. In addition, non-resonant low field absorption may have a contribution related to the high conductivity of metallic microparticles, as it has been previously observed for different kinds of magnetic powders [41]. However, we believe that a detailed study of the physical origin of the lower-frequency maximum is a separate complex task that is beyond the scope of this work.

### 3.2. Estimation of the Curie Temperature of Composites Based on Microwave Measurements

There are various experimental methods for determining the Curie temperature of ferromagnetic materials (see, for example, [12,43]). In particular, the Curie temperature can be found from the analysis of the temperature dependences of electric resistivity, magnetization, initial permeability and heat capacity. Other methods to determine the Curie temperature include differential scanning calorimetry, the Mössbauer technique and neutron scattering. However, these methods require complex experimental equipment. It should be noted that the Curie temperature values obtained for the same ferromagnetic material by various methods may differ significantly due to remnants of spontaneous magnetization above the Curie temperature, sample purity, etc. [11,12]. In this work, the Curie temperature was determined from the inflection point of the temperature dependence of the composite static permeability [44].

**Figure 6:**
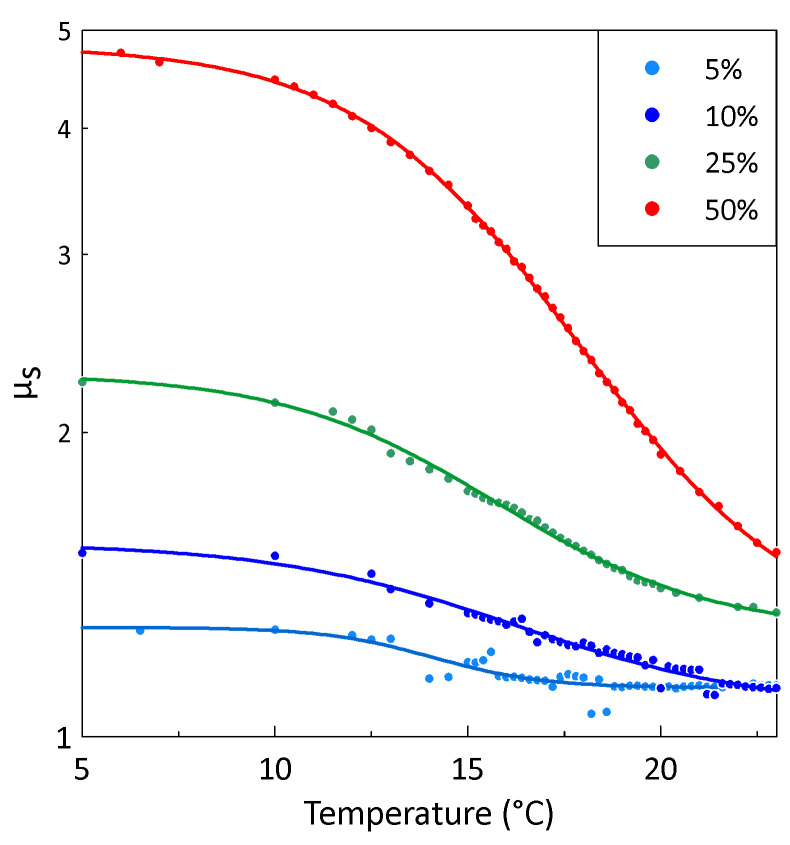
Temperature dependence of the composite static permeability *μ*_s_ for different volume fractions of gadolinium particles (indicated in the legend). Symbols represent experimental data; lines correspond to the fitting by Equation (2).

For each temperature and volume fraction of gadolinium, the static permeability of the composite was determined as the median value in the frequency range of 100 to 200 MHz. The obtained temperature dependence of the composite static permeability *μ*_s_ for different volume fractions of gadolinium particles is shown in Figure 6. The static permeability decreases gradually with increasing temperature. Significant changes in the composite static permeability takes place in the temperature transition range of 10 to 20 °C. The Curie temperature can be defined as the inflection point of the temperature dependence of *μ*_s_(*t*) [43,44].

In order to determine the inflection point, the dependence of *μ*_s_(*t*) was fitted by the Boltzmann sigmoid function, which describes a smooth transition between two states:

(2) $$\mu_{s}(t)=\mu_{2}+\frac{\mu_{1}-\mu_{2}}{1+\exp[(t_{0}-t)/\Delta t]}.$$

Here, *μ*_1_ and *μ*_2_ are the values of the static permeability at *t* → ±∞, and the transition between two states is characterized by the central temperature *t*_0_ and the temperature range width Δ*t*.

**Figure 7:**
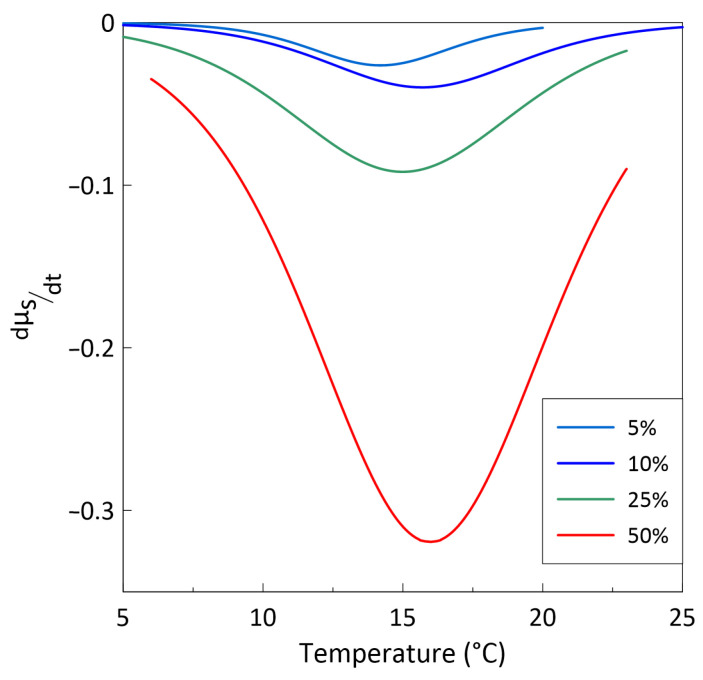
The calculated dependence of d*μ*_s_/d*t* on temperature for different volume fractions of gadolinium particles (indicated in the legend). The Curie temperature corresponds to the minimum of d*μ*_s_/d*t*.

The fitting results are presented in Figure 6 by solid lines. Using Equation (2), the temperature dependence of d*μ*_s_/d*t* was calculated for different volume fractions of gadolinium particles (see Figure 7). The Curie temperature corresponds to the minimum of the temperature dependence of d*μ*_s_/d*t*.

The calculated Curie temperatures of all composites are 15 ± 0.5 °C (288 K) and are practically independent of the gadolinium volume fraction (see Figure 7). As the calculated Curie temperatures *T*_C_ of all composites are close to the same value and are practically independent of the gadolinium volume fraction, it seems that the obtained *T*_C_ represents the true Curie temperature of the filler. The obtained Curie temperature of the composites is several degrees lower than that for bulk gadolinium [11]. This discrepancy may be due to the presence of a small amount of impurities in the powders, mechanical stresses and a surface-related oxide layer covering the gadolinium particles [35].

### 3.3. Intrinsic Permeability of Filler Particles

To retrieve the intrinsic permeability of the filler, mixing rules are usually used. Mixing rules are algebraic equations that relate the constitutive parameters (permittivity and permeability) of a composite to those of its components and their volume fractions. Currently, more than 50 mixing rules are known in the literature [45,46,47,48]. In this study, to retrieve the intrinsic permeability of the filler particles, we selected a mixing rule proposed by Odelevsky [49]. It has been shown previously that the Odelesky mixing rule provides good agreement with experimental data for composites with different ferromagnetic fillers [50,51,52]. The modified Odelevsky mixing rule takes into account the shape of the filler particles and the percolation threshold [46]. The percolation threshold is the critical amount of filler at which the composite begins to conduct DC current and the effective permittivity of the composite tends to infinity.

According to the Odelevsky mixing rule, the permittivity and permeability of a composite can be described by the following equations:

(3) $$\varepsilon =\varepsilon_{m}\left[1+\frac{p}{N(1-p/p_{c})}\right],$$

(4) $$\mu =1+\frac{p(\mu_{in}-1)}{N(\mu_{in}-1)(1-p/p_{c})+1}.$$

Here, the subscripts *m* and *in* denote the matrix and filler, respectively; *N* is the depolarization (demagnetization) factor depending on the filler particle shape; *p* is the volume fraction of the filler and *p_c_* is the percolation threshold. Note that Equations (3) and (4) are valid at *p* < *p_c_*, and we take into account that *ε*_*m*_/*ε*_*in*_ << 1 and *μ_m_* = 1.

Equations (3) and (4) have two free parameters: the depolarization (demagnetization) factor of the particles *N* and the percolation threshold *p_c_*. The Odelevsky mixing rule reduces to the Bruggeman effective medium theory when *p_c_* = *N* and to the Maxwell Garnett mixing rule when *p_c_* = 1 [46].

**Figure 8:**
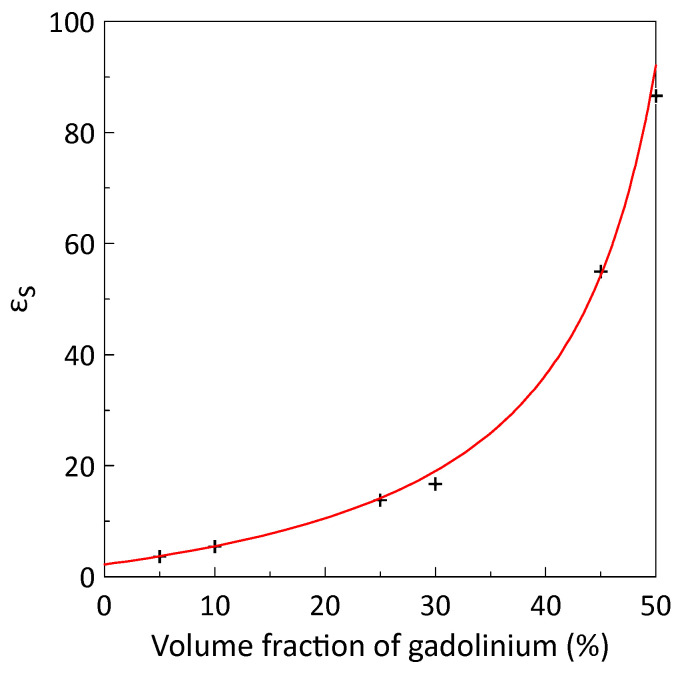
The dependence of the static permittivity *ε*_s_ on the volume fraction of gadolinium particles. Symbols represent experimental data; the line represents fitting using Equation (3). The parameters used for fitting are *ε_m_* = 2.2, *p_c_* = 0.58 and *N* = 0.08.

Figure 8 shows the measured dependence of the composite static permittivity *ε*_s_ on the volume fraction of filler particles. The solid line represents the fitting of the experimental data using Equation (3) with the permittivity of wax *ε_m_* = 2.2. From the fitting, we found that *p_c_* = 0.58 and *N* = 0.08. The obtained value of the depolarization (demagnetization) factor *N* shows that the gadolinium particles in the composite have a stone-like shape, which is related to the powder production method.

Using the values of the percolation threshold *p_c_* and depolarization factor *N* obtained from the dependence of the static composite permittivity on the gadolinium volume fraction, we can calculate the intrinsic permeability of the filler. The intrinsic permeability *μ_in_* can be expressed from Equation (4) as follows:

(5) $$\mu_{in}=1+\frac{\mu -1}{p-N(\mu -1)(1-p/p_{c})}.$$

The procedure for obtaining the frequency dependence of the intrinsic permeability of the filler can be described as follows. At a fixed temperature, the intrinsic permeability was calculated using Equation (5) for each value of the gadolinium volume fraction. Then, the obtained frequency dependences of the intrinsic permeability were fitted by Equation (1). For a set of approximated dependences at different volume fractions of gadolinium in composites, a median dependence corresponding to the intrinsic permeability of the particles was obtained.

**Figure 9:**
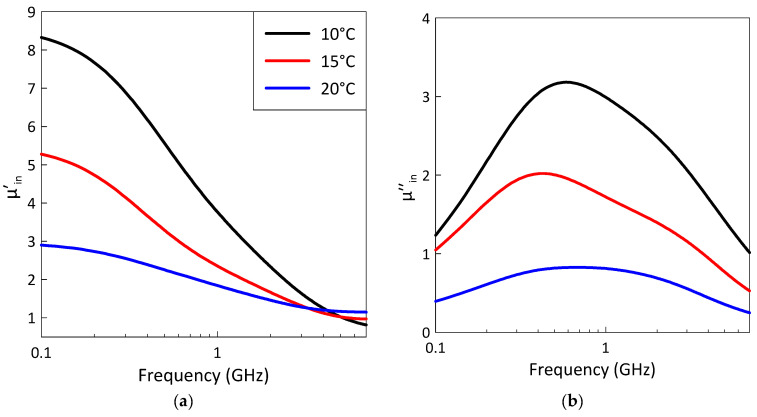
The retrieved frequency dependences of the real (**a**) and imaginary (**b**) parts of the intrinsic permeability of filler particles at different temperatures. The legend describing the different temperatures is the same for parts (**a**,**b**).

Figure 9 shows the retrieved frequency dependences of the intrinsic permeability of the filler particles at three temperatures, 10, 15 and 20 °C. The peak of the imaginary part of the intrinsic permeability also consists of two maxima corresponding to microwave absorption phenomena associated with the magnetic domain structure and FMR.

The intrinsic permeability is preserved even at 20 °C, which is above the Curie temperature (see Figure 9). This is because the ferromagnetic–paramagnetic phase transition in the studied composites takes place over a wide temperature range (see Figure 6). The appearance of a wide transition temperature range can be explained by the existence of magnetic clusters near the Curie temperature [53,54]. The temperature effect on the frequency dependence of the permeability in composites filled with gadolinium particles may be interpreted using the previously developed cluster magnetization model [35,55].

**Figure 10:**
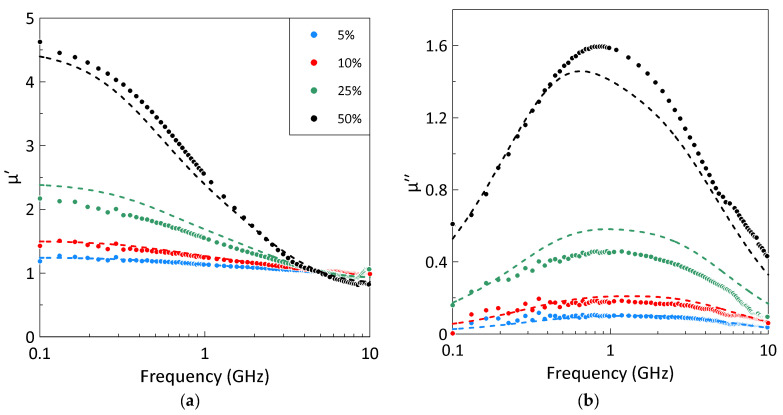
The frequency dependences of the real (**a**) and imaginary (**b**) parts of the composite permeability at 10 °C for different volume fractions of gadolinium particles. Symbols represent experimental data; lines correspond to the results of calculations using the obtained intrinsic permeability of filler particles. The legend describing the different gadolinium volume fractions is the same for parts (**a**,**b**).

To test the accuracy of the used Odelevsky mixing rule, the permeability of composites at different volume fractions of gadolinium particles was calculated from the obtained frequency dependence of the intrinsic permeability using Equation (4). Figure 10 shows a comparison of experimental data on the frequency dependence of the composite permeability at a temperature of 10 °C with the calculated results. At low volume fractions of gadolinium, the calculated permeability values are in a good agreement with experimental data, whereas at higher filler concentrations, a significant discrepancy is observed. This discrepancy can be explained as follows. First, there is a noticeable inaccuracy in the obtained intrinsic permeability values due to the averaging procedure used. Second, the Odelevsky mixing rule yields considerable inaccuracies in determining the constitutive parameters of the composite when the filler concentration is close to the percolation threshold *p_c_* [46]. In the composites studied, the maximum volume fraction of gadolinium particles is 0.5, and *p_c_* = 0.58.

Due to the high temperature sensitivity of the composite permeability, the studied composite materials filled with gadolinium powder may be promising for the development of temperature-tunable microwave screens and sensors.

## 4. Conclusions

The microwave permeability of composites with paraffin wax matrix filled with gadolinium powder was studied. The frequency dependence of the imaginary part of the permeability for all composites exhibited a broad maximum. The analysis showed that magnetic losses consist of two resonances. Both the position and the magnitude of these resonances depend on the volume fraction of gadolinium particles, the external magnetic field and the temperature. An increase in the volume fraction of gadolinium particles enhances the relative magnitude of the lower-frequency maximum. The application of an external static magnetic field up to 800 Oe changes the shape of the magnetic loss peak. The position of the lower-frequency maximum shifts towards higher frequencies and its magnitude decreases. The higher-frequency maximum also shifts towards higher frequencies but maintains a nearly constant magnitude. The lower-frequency maximum corresponds to resonance phenomena associated with low field non-resonant absorption, whereas the higher-frequency maximum is attributed to FMR.

The Curie temperature of the composites was obtained from the temperature dependence of the static permeability. The Curie temperature was found to be 15 ± 0.5 °C and was independent of the gadolinium volume fraction. This value is only about 5 °C lower than the Curie temperature for bulk gadolinium. It was observed that a ferromagnetic–paramagnetic phase transition in the studied composites takes place over a temperature range of about 10 °C.

To retrieve the intrinsic permeability of the filler particles, the Odelevsky mixing rule was used. The mixing rule accurately fits the experimental data on the dependence of the static permittivity on the gadolinium volume fraction. Calculations using the Odelevsky mixing rule give the values of the percolation threshold *p_c_* = 0.58 and the depolarization factor *N* = 0.08. The depolarization factor value corresponds to a stone-like shape of the particles. For the first time, the frequency dependence of the intrinsic permeability of gadolinium particles at different temperatures was calculated, and it was found that the frequency dependence of the intrinsic permeability can be fitted by a sum of two Lorentzian terms.

## Data Availability

The original contributions presented in this study are included in the article. Further inquiries can be directed to the corresponding author.
